# Genotype-phenotype correlation in XLHED: insights into the biology of ectodysplasin

**DOI:** 10.1186/1746-160X-8-S1-P1

**Published:** 2012-05-25

**Authors:** KM Huttner, DK Grange, OD Klein, H Schneider

**Affiliations:** 1 Edimer Pharmaceuticals Inc, Cambridge, USA; 2 Washington University, St Louis, USA; 3 University of California, San Francisco, USA; 4 University Hospital Erlangen, Germany

## 

X-linked hypohidrotic ectodermal dysplasia (XLHED) is the most common of the ectodermal dysplasias, with a classic presentation of hypodontia, hypohidrosis, hypotrichosis and secretory gland hypoplasia. Mutations in the ectodysplasin gene (*EDA*) underlie XLHED with nearly 200 different mutations reported. *EDA* encodes a type 2 transmembrane protein of the TNF family (EDA-A1), the active form of which is released from the cell surface following furin proteolytic cleavage. During normal human development, EDA-A1 multimers bind to their cognate receptor (EDAR) driving maturation of ectodermal placodes into sweat ducts, hair follicles, tooth buds, and secretory glands. In the absence of functional EDA-A1, all of the above are compromised.

As genotype-phenotype correlations in XLHED have not yet been characterized satisfactory, natural history studies incorporating non-invasive, quantitative assessments were conducted on 120 genotyped XLHED males, age newborn to 60 years. This extensive cohort, representing 69 different mutations, can now be analyzed for phenotypic variation associated with alterations in the intracellular, transmembrane, extracellular pre-cleavage, furin recognition, collagen-like domain, and receptor binding regions of the EDA-A1 protein. Approximately 3/4 of the XLHED patients evaluated had missense or nonsense *EDA* mutations and 1/4 had indel mutations. In an initial approach to genotype-phenotype correlation, the severe or “null” phenotype (anhidrosis), with absence of both sweat ducts and inducible sweating, was associated with 55 of the *EDA* genotypes. The remaining 14 *EDA* mutations were associated with the presence of normal appearing but hypofunctional sweat ducts, highlighting specific regions of the EDA-A1 protein where non-termination mutations allowed for activation of sweat duct development but through aberrant pathways. These experiments of nature may provide novel insights into the biology of ectodysplasin biosynthesis and functional activation of the ectodysplasin/EDAR/NFkB pathway.

